# Supplementary material for: Insights into the functional and genetic basis of heteranthery in Arthrostemma ciliatum Pav. ex D.Don (Melastomataceae)
Source: BMC Plant Biol. 2026 Jun 16;26:1045. doi: 10.1186/s12870-026-09093-6 (PMC13273962; doi:10.1186/s12870-026-09093-6)
Supplement: Supplementary file 1 — Appendix 1: Sample names and summary table of RNA extractions. Appendix-2: Developmental series images of flower buds and stamen. Appendix-3: MultiQC reports of raw data; after trimming and after removal of rRNA. Appendix-4: Scatterplot of transformed counts from two samples. Appendix-5: DESeq results tables with differentially expressed genes of interest from each comparison showing FDR, LFC, Counts and annotations. Appendix-6: Violin plots of theca size by stamen type and flower. Appendix-7: Violin plots of length of anthers used for pollen counts and pollen numbers by stamen type. Appendix-8: Violin plots of pollen size by stamen type and flower. Appendix-9: Violin plots of pollen germination by stamen types and flower. [file 12870_2026_9093_MOESM1_ESM.zip › Appendix-1_RNA_extraction_summary_table.pdf]

Table 1: Sample table for *Arthrostemma ciliatum.* Sample names are indicative of the species (AC), stamen whorl (O-outer/antesepalous and I-inner/antepetalous) and sampling event number. The Condition column for each sample refers to the stamen whorl and the developmental stage at which it was sampled. RINe refers to RNA Integrity Number equivalent output by the Agilent Tape station analysis, a value of 10.0 indicates high RNA integrity and minimal degradation. Conc refers to RNA concentration after isolation obtained by the Agilent Tapestation analysis. VolEl refers to the final volume of elution.

Sample name	No. of flowers/ stamen	Condition (whorl+ bud size)	Date	Sampling time (24h)	Size of buds	RNA extraction kit	RINe value	RNA Conc (ng/µl)	VolEl (µL)
ACO 27	12 /48	O3	21.09.2023	10:07-11:10	2-3mm	RNeasy Plus Micro	10.0	90.5	20
ACI 27	12 /48	I3	21.09.2023	10:07-11:10	2-3mm	RNeasy Plus Micro	10.0	67.4	20
ACO 24	12 /48	O3	11.09.2023	10:31-11:38	2-3mm	RNeasy Plus Micro	10.0	108	14
ACI 24	12 /48	I3	11.09.2023	10:31-11:38	2-3mm	RNeasy Plus Micro	10.0	41.3	14
ACO 25	12 /48	O3	12.09.2023	10:00-10:51	2-3mm	RNeasy Plus Micro	10.0	62.3	20
ACI 25	12 /48	I3	12.09.2023	10:00-10:51	2-3mm	RNeasy Plus Micro	10.0	95.1	20
ACO 28	8 /32	O5	21.09.2023	11:11-11:43	5-5.5mm	RNeasy Plus Micro	9.7	263	30
ACI 28	8 /32	I5	21.09.2023	11:11-11:43	5-5.5mm	RNeasy Plus Micro	9.8	273	30
ACO 23	12 /48	O5	07.09.2023	10:01-11:07	5-5.5mm	RNeasy Plus Micro	6.8*	368	14
ACI 23	12 /48	I5	07.09.2023	10:01-11:07	5-5.5mm	RNeasy Plus Micro	6.8*	387	14
ACO 26	10 /40	O5	12.09.2023	10:55-11:42	5-5.5mm	RNeasy Plus Micro	9.9	305	30
ACI 26	10 /40	I5	12.09.2023	10:55-11:42	5-5.5mm	RNeasy Plus Micro	9.8	267	30
ACO 15	3 /12	O12	26.07.2023	10:58-11:14	11-12mm	RNeasy Plant mini	9.8	51.6	30
ACI 15	3 /12	I12	26.07.2023	10:58-11:14	11-12mm	RNeasy Plant mini	8.8	40.2	30
ACO 17	3 /12	O12	31.07.2023	11:23-11:42	11-12mm	RNeasy Plant mini	9.5	42.7	30
ACI 17	3 /12	I12	31.07.2023	11:23-11:42	11-12mm	RNeasy Plant mini	9.5	45	30
ACO 21	3 /12	O12	30.08.2023	10:25-10:39	11-12mm	RNeasy Plant mini	9.9	69.6	30
ACI 21	3 /12	I12	30.08.2023	10:25-10:39	11-12mm	RNeasy Plant mini	9.7	39.8	30
ACO 13	3 /12	O18	25.07.2023	10:48-10:58	17-18mm	RNeasy Plant mini	9.3	221	30
ACI 13	3 /12	I18	25.07.2023	10:48-10:58	17-18mm	RNeasy Plant mini	9.1	169	30
ACO 14	3 /12	O18	26.07.2023	10:37-10:52	17-18mm	RNeasy Plant mini	8.7	175	30
ACI 14	3 /12	I18	26.07.2023	10:37-10:52	17-18mm	RNeasy Plant mini	9.7	123	30
ACO 16	3 /12	O18	31.07.2023	11:06-11:22	17-18mm	RNeasy Plant mini	10.0	316	30
ACI 16	3 /12	I18	31.07.2023	11:06-11:22	17-18mm	RNeasy Plant mini	9.9	311	30

*RINe value lower than true value due to excessive amounts of RNA used during measurement. The values improved to above 9.0 upon dilution
