# Supplementary figures and images for: Insights into the functional and genetic basis of heteranthery in Arthrostemma ciliatum Pav. ex D.Don (Melastomataceae)
Source: BMC Plant Biol. 2026 Jun 16;26:1045. doi: 10.1186/s12870-026-09093-6 (PMC13273962; doi:10.1186/s12870-026-09093-6)

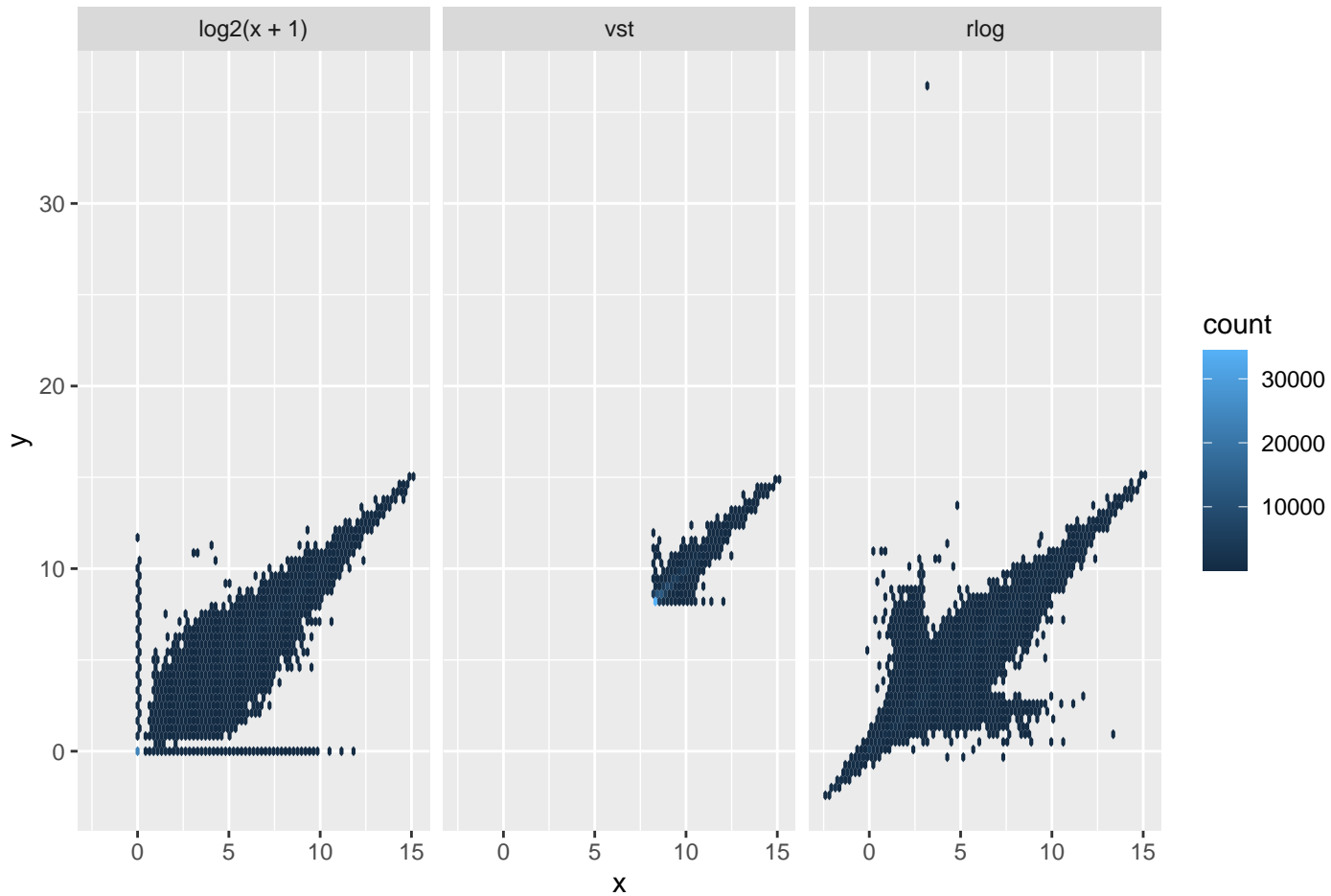

Supplement: Supplementary file 1 — Appendix 1: Sample names and summary table of RNA extractions. Appendix-2: Developmental series images of flower buds and stamen. Appendix-3: MultiQC reports of raw data; after trimming and after removal of rRNA. Appendix-4: Scatterplot of transformed counts from two samples. Appendix-5: DESeq results tables with differentially expressed genes of interest from each comparison showing FDR, LFC, Counts and annotations. Appendix-6: Violin plots of theca size by stamen type and flower. Appendix-7: Violin plots of length of anthers used for pollen counts and pollen numbers by stamen type. Appendix-8: Violin plots of pollen size by stamen type and flower. Appendix-9: Violin plots of pollen germination by stamen types and flower. [file 12870_2026_9093_MOESM1_ESM.zip › Appendix-4_scatterplot_count_transformations_noleaf.pdf]

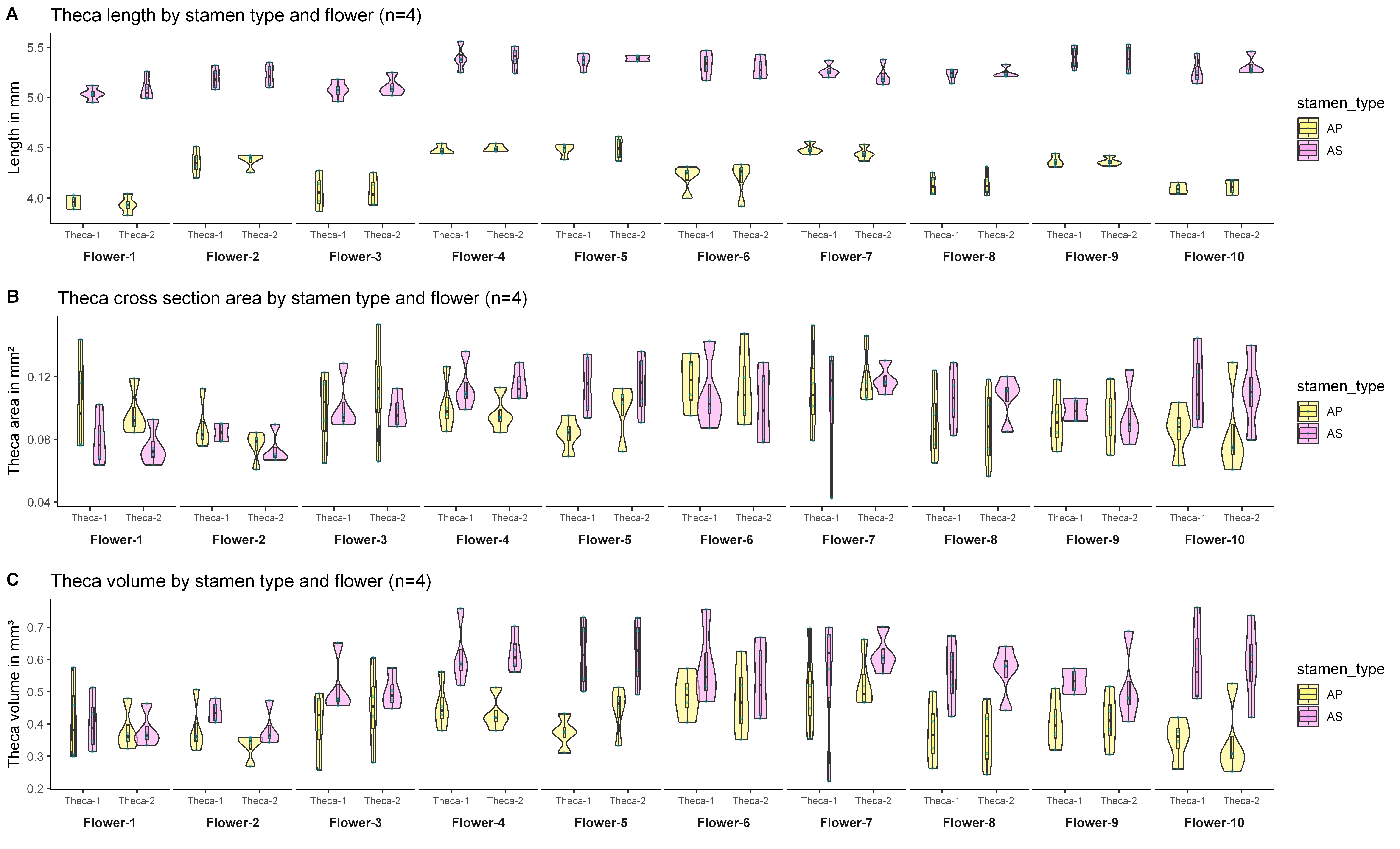

Supplement: Supplementary file 1 — Appendix 1: Sample names and summary table of RNA extractions. Appendix-2: Developmental series images of flower buds and stamen. Appendix-3: MultiQC reports of raw data; after trimming and after removal of rRNA. Appendix-4: Scatterplot of transformed counts from two samples. Appendix-5: DESeq results tables with differentially expressed genes of interest from each comparison showing FDR, LFC, Counts and annotations. Appendix-6: Violin plots of theca size by stamen type and flower. Appendix-7: Violin plots of length of anthers used for pollen counts and pollen numbers by stamen type. Appendix-8: Violin plots of pollen size by stamen type and flower. Appendix-9: Violin plots of pollen germination by stamen types and flower. [file 12870_2026_9093_MOESM1_ESM.zip › Appendix-6_Theca_size_comparisons_between_stamen_types_by_flower.png]

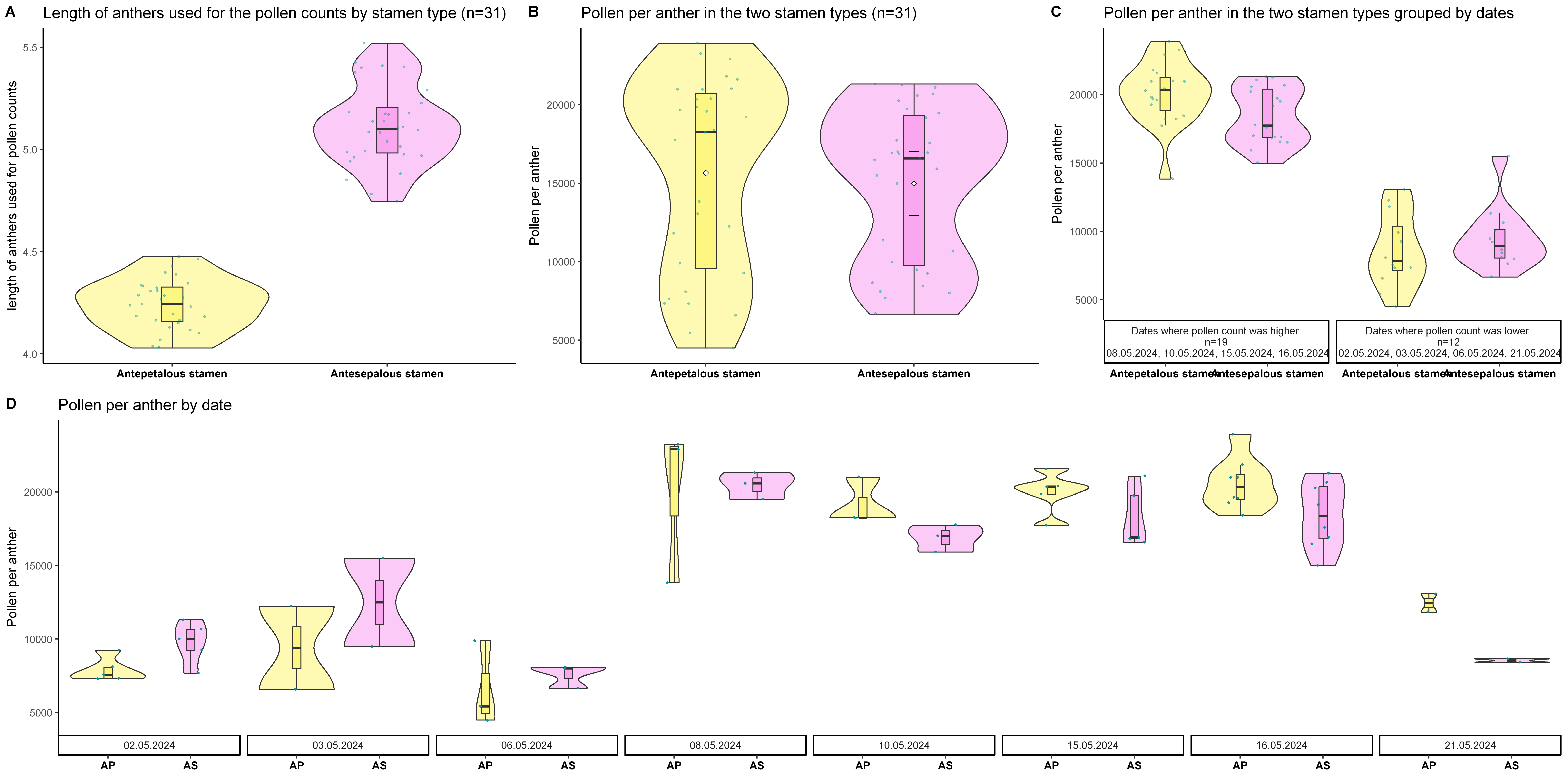

Supplement: Supplementary file 1 — Appendix 1: Sample names and summary table of RNA extractions. Appendix-2: Developmental series images of flower buds and stamen. Appendix-3: MultiQC reports of raw data; after trimming and after removal of rRNA. Appendix-4: Scatterplot of transformed counts from two samples. Appendix-5: DESeq results tables with differentially expressed genes of interest from each comparison showing FDR, LFC, Counts and annotations. Appendix-6: Violin plots of theca size by stamen type and flower. Appendix-7: Violin plots of length of anthers used for pollen counts and pollen numbers by stamen type. Appendix-8: Violin plots of pollen size by stamen type and flower. Appendix-9: Violin plots of pollen germination by stamen types and flower. [file 12870_2026_9093_MOESM1_ESM.zip › Appendix-7_Pollen_number_plots_1.png]

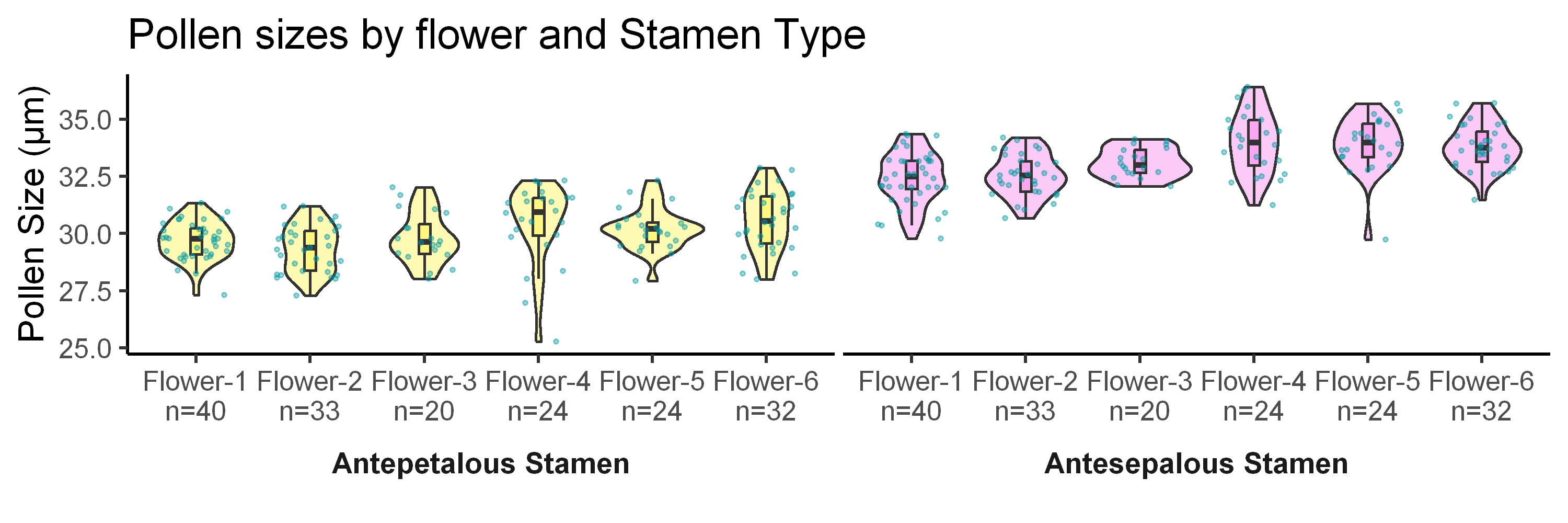

Supplement: Supplementary file 1 — Appendix 1: Sample names and summary table of RNA extractions. Appendix-2: Developmental series images of flower buds and stamen. Appendix-3: MultiQC reports of raw data; after trimming and after removal of rRNA. Appendix-4: Scatterplot of transformed counts from two samples. Appendix-5: DESeq results tables with differentially expressed genes of interest from each comparison showing FDR, LFC, Counts and annotations. Appendix-6: Violin plots of theca size by stamen type and flower. Appendix-7: Violin plots of length of anthers used for pollen counts and pollen numbers by stamen type. Appendix-8: Violin plots of pollen size by stamen type and flower. Appendix-9: Violin plots of pollen germination by stamen types and flower. [file 12870_2026_9093_MOESM1_ESM.zip › Appendix-8_Pollensizes_1.png]

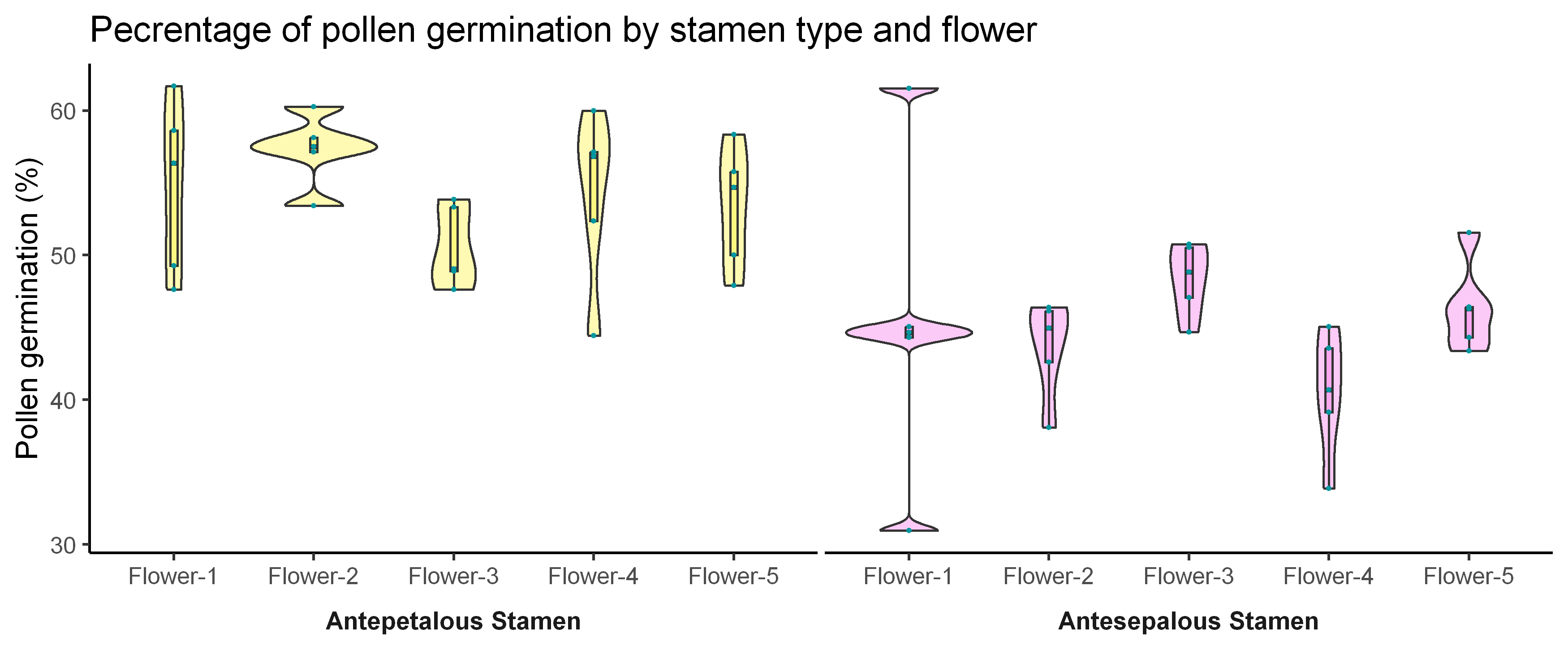

Supplement: Supplementary file 1 — Appendix 1: Sample names and summary table of RNA extractions. Appendix-2: Developmental series images of flower buds and stamen. Appendix-3: MultiQC reports of raw data; after trimming and after removal of rRNA. Appendix-4: Scatterplot of transformed counts from two samples. Appendix-5: DESeq results tables with differentially expressed genes of interest from each comparison showing FDR, LFC, Counts and annotations. Appendix-6: Violin plots of theca size by stamen type and flower. Appendix-7: Violin plots of length of anthers used for pollen counts and pollen numbers by stamen type. Appendix-8: Violin plots of pollen size by stamen type and flower. Appendix-9: Violin plots of pollen germination by stamen types and flower. [file 12870_2026_9093_MOESM1_ESM.zip › Appendix-9_Pollen_germination_by_StamenType_and_flower.png]
